# A Personalized Approach to Evaluating and Treating Autism Spectrum Disorder

**DOI:** 10.3390/jpm12020147

**Published:** 2022-01-24

**Authors:** Richard E Frye, Shannon Rose, Richard G. Boles, Daniel A. Rossignol

**Affiliations:** 1 Section on Neurodevelopmental Disorders, Barrow Neurological Institute at Phoenix Children’s Hospital, Phoenix, AZ 85016, USA; 2 Department of Child Health, University of Arizona College of Medicine—Phoenix, Phoenix, AZ 85004, USA; 3 Arkansas Children’s Research Institute, Little Rock, AR 72202, USA; SROSE@uams.edu; 4 Department of Pediatrics, University of Arkansas for Medical Sciences, Little Rock, AR 72205, USA; 5 NeurAbilities Healthcare, Voorhees, NJ 08043, USA; rboles@neurabilities.com; 6 Rossignol Medical Center, 24541 Pacific Park Drive, Suite 210, Aliso Viejo, CA 92656, USA; rossignolmd@gmail.com

The most recent Center for Disease Control and Prevention estimates suggest that 1 in every 44 children (>2%) in the United States (US) is affected by autism spectrum disorder (ASD). Despite decades of research, it is difficult to identify ASD early in infancy when treatments are likely to be most effective. Recent evidence suggests that optimal outcomes for individuals with ASD may be better achieved by substantially improving our understanding of the underlying biological mechanisms or causes of ASD and from identifying effective treatments. One of the limitations for understanding ASD in greater depth is the well-known heterogeneity in clinical presentation, genes and pathways involved, co-morbid medical conditions, and response to treatment. Many have suggested that ASD is best represented as a set of distinct subgroups known as ‘Autisms’ rather a spectrum with a hereunto undefined number of dimensions, but little evidence points to one or the other approach as being the most valid. Thus, a personalized medicine approach may be optimal for understanding and treating each individual with ASD based on their individual unique characteristics.

Regardless of whichever approach is best, the fact remains that patients with ASD are all unique in many ways and require a personalized approach to their care. In this sense, we developed this Special Issue in order to start to better understand ASD by using a personalized medicine approach so that an eventual plan for understanding and treating patients with ASD can be developed as we go forward into the future. The Special Issue published 21 articles with 8 research articles, 10 review articles and 3 case studies. Of these articles, 14 discussed different underlying phenotypes of ASD, 12 discussed evidence for potential promising treatments, two described prenatal factors that might influence the development of ASD, and one described an alternative approach to understanding social interactions to improve diagnosis.

One major limitation to identifying and treating children with ASD is the diagnostic tests used. One of the current gold-standard diagnostic tests, the Autism Diagnostic Observation Schedule (ADOS), uses a trained examiner to elicit social ‘presses’ designed to evoke social interactions in the patient. A paper from the group at Rutgers University uses the standard ADOS examination to expand on an analysis of social interactions [1]. The group advances the examination by postulating that social interactions are not one-sided but involve a social dyad of interacting nervous systems processing sensory-motor information. Using wearable devices to digitally monitor movements, this group analyzes behaviors at both micro and macro levels with non-linear dynamic equations to derive advanced metrics of social interaction such as strength, coherence and variability of the dyad interactions. As such, this paper provides an interesting conceptual advance by both uncovering an underlying limitation of current approaches of evaluating social interactions and expands the use of such instruments to improve diagnosis by integrating a more complex analysis of the underlying dynamics of the nervous system.

The etiology of ASD is unknown in many, perhaps the majority, of cases. An increasing number of studies have pointed to genetic vulnerabilities interacting with environmental factors to trigger the development of ASD. It is being increasingly recognized that one of the most influential environments that will shape an individual’s life is the prenatal maternal environment. This research is particularly insightful as it not only points to potential pathophysiological factors that cause ASD but also provides insight into potential strategies for preventing ASD from developing in the first place. Using a large dataset of medical claims across 123,824 pregnancies, researchers from the Rensselaer Polytechnic Institute identified pregnancy factors, which both increased and decreased the risk of developing ASD [2]. A common shortfall of research on prenatal factors is that most studies are associational rather than mechanistic. In this Special Issue, a group of researchers who have recently linked specific prenatal environmental exposures, including air pollution and nutritional metals, to long-term changes in mitochondrial function provide a comprehensive review of evidence linking prenatal factors that have been previously epidemiologically linked to ASD with mitochondrial dysfunction, thus providing a potential mechanism for these prenatal influences [3]. Connections with oxidative stress and disruption of the immune system are also outlined.

One of the most rational methods of approaching the management of individuals with ASD is to define subgroups. Fourteen papers describe various potential subgroups. This set of papers illustrates multiple viewpoints and techniques that can be used to define subgroups. Two papers describe methods for stratifying subgroups. A group of leaders in the treatment of ASD in Argentina describe their approach for defining subgroups by concentrating on the diagnosis and treatment of common concomitant medical problems and propose a classification system based on responses to the treatment of these concomitant problems [4]. A nice overview from a group in Qatar outlines the potential for biomarker use in the stratification of ASD [5]. This paper provides nice illustrations of the various biomarkers and approaches as well as provides some limited examples of the biomarkers described.

One important subgroup of patients with ASD that can be rather rigorously defined consists of those with known genetic changes. One article reported females with potential causative copy number changes on the chromosomal microarray. Variants involved in synaptic structure and transmission were most prominent, although other pathways including ion channels, neuron projection, and mRNA/protein processing, all mechanisms previously linked to ASD, were also identified. An interesting exploratory analysis found significantly lower restrictive and repetitive behaviors in those with presumed disease causative copy number variations as compared to females without such genetic changes [6]. One of the mysteries of ASD is the high heritability index without a high rate of common causative inherited genetic mutations. This is potentially due to several phenomena and historically has often been attributed to extreme genetic heterogeneity with multiple rare variants in many genes being causal for ASD. Another potential explanation is the complex interaction of genetic variations which by themselves may not be severe enough to express the ASD phenotype but together may result in disease. By analyzing the genome of a well-known professor with high-functioning ASD, Dr Temple Grandin, insight into how interactions between a complexity of genetic variations may result in the ASD phenotype is discussed in one article [7]. In another article, a genomic clinical decision support tool that focuses on relatively common DNA variants in pathways that can be targeted for treatment to improve symptoms is described along with several cases successfully treated using this tool [8]. In many cases, the complexity of genetic changes (as well as potential environmental interactions) is not clear. In a nice article, the overlap between ASD and Ehlers-Danlos syndromes and hypermobility spectrum disorders (EDS/HSD) is discussed [9]. This latter set of disorders also is believed to have an inherited component, which, similarly to ASD, remains elusive in many cases. Hypermobility spectrum disorders are being increasing recognized in ASD, in addition to comorbidities associated with both disorders including autonomic and immune dysregulation.

Two articles discuss aspects of the immune phenotype related to ASD. By conducting a meta-analysis on the literature, evidence for two specific subtypes of immunoglobulin dysregulation, low total IgG, and elevations in IgG4 are found [10]. Other phenotypes such as those with immune deficiencies as well as those with autoimmune encephalopathies are also described. In another article, the importance of mast cell activation in activating microglia resulting in focal brain inflammation and disrupted synaptic pruning is discussed [11]. Similar to EDS/HSD, aberrant mast cell activation and related symptomatology are common in cohorts with ASD and may designate endophenotypes of diseases.

Metabolic subtypes of ASD are also being recognized. One article describes a potential important subtype of ASD with a unique type of mitochondrial dysfunction, which may be related to environmental exposures [3]. In another article, researchers from Italy and France demonstrate that children with ASD who had elevations in acyl-carnitines in their blood exhibit changes in fatty-acid oxidation and electron transport chain complex activity in their fibroblasts [12]. Another article describes neurotransmitter dysregulation associated with mitochondrial disease in a unique patient [13]. Most interestingly, these changes in central neurotransmitters were discovered non-invasively by using resting state functional magnetic resonance imaging (rsfMRI) and confirmed by examination of the cerebrospinal fluid. Another study examined fecal metabolites from individuals with ASD and gastrointestinal symptoms, demonstrating that a model with five metabolites provides excellent separation between the ASD and a typically developing control group [14].

Two articles describe an increasingly recognized subgroup of children with ASD who have a unique metabolic-immune disorder that may influence their brain development both prenatally and postnatally. Autoantibodies that bind the folate receptor alpha (FRα), the major transporter of folate into the brain and across the placenta, are found in children with ASD as well as their parents. One article discusses FRα autoantibodies in a broad context, discussing the overlap with cerebral folate deficiency (CFD) and neural tube defects and a subgroup of ASD patients who may be more severely affected due to both prenatal and postnatal autoantibody exposure [15]. Another article conducts a meta-analysis on the available data to provide more concrete prevalence values of FRα autoantibodies in children with ASD and their families [16]. Interestingly, the prevalence rates suggest that FRα autoantibodies have a familial component, although the mechanism of this is not well understood and may be polygenic with environmental influences. This latter article also provides a meta-analysis to better define the overlap between children with ASD and those with CFD.

Two papers discuss regressive-type ASD, which may constitute yet another endophenotype of ASD. One paper describes a unique type of mitochondrial dysfunction that might underlie metabolic disturbance in children with ASD and neurodevelopmental regression [3]. In the other article, researchers at Barrow Neurologic Institute at Phoenix Children’s Hospital use rsfMRI to demonstrate that children with regressive-type ASD appear to have intact cognitive networks that potentially cannot be expressed because of aberrant interfering networks, essentially creating a locked-in network syndrome [17].

Eleven papers provide either original data of treatment effectiveness or review the current literature of potentially effective treatments. Five papers describe treatments addressing specific metabolic defects. Building on the subgroup of children with FRα autoantibodies, one paper provides insight into the potential prenatal and postnatal treatment to prevent and treat ASD [15], while a second paper uses meta-analysis to document evidence for effectiveness and efficiency of *d,l*-leucovorin, the major treatment for children with ASD and FRα autoantibodies, across published clinical trials [16]. In another systematic review and meta-analysis, the effectiveness of cobalamin, a treatment that targets dysfunctional methylation and redox pathways, is analyzed, along with an examination of its metabolic effects [18]. Researchers from Italy and France demonstrate that resveratrol may be effective in correcting metabolic defects in fibroblasts from children with ASD and fatty-acid oxidation defects [12]. Lastly, researchers from Arizona State University demonstrate that Microbiota Transfer Therapy appear to normalize abnormal fecal metabolites in a group of people with ASD [14].

Two papers discuss potential treatments for immune dysfunction in ASD. Using a systematic review and meta-analysis approach, evidence for the effectiveness of intravenous immunoglobulin (IVIG) in ASD is presented [10], while another study discusses approaches to treating mast cell disorders with an emphasis on luteolin [11].

Two papers examine general treatments for children with ASD. In a large national survey of 1286 participants across the US, several nutraceuticals, including folate and cobalamin, were found to be rated by families to have greater benefits than a similar survey of psychiatric and seizure medications [19]. Another study demonstrates how examining genetic variations can help consider targeting common nutraceutical treatments on a personalized basis [8].

Transdermal Electrical Neuromodulation (TEN) is an approved safe non-invasive treatment for several disorders including attention-deficit hyperactivity disorder and migraines. In the first study using TEN to treat individuals with ASD, researchers at the Barrow Neurologic Institute at Phoenix Children’s Hospital demonstrate the feasibility and potential effectiveness of TEN treatment for anxiety as well as demonstrate how autonomic system biomarkers may be used to predict response to TEN treatment [20].

Lastly, in an important and enlightening systematic review, evidence is presented that parental involvement in therapy relates to improved parental quality of life [21].

We believe that this collection of articles provides insight into the current state of ASD diagnosis, evaluation, and treatment using a personalized medicine approach. While each of these articles individually advances this goal, we believe that this collection fits in with the current understanding of ASD and advances this understanding. Perhaps most apparent, these articles underline the primacy of mitochondria in ASD, including oxidative stress and redox regulation, which are themselves major functions of mitochondria. Although the connection between ASD and this important cellular organelle is hardly new, this collection of papers serves to demonstrate the ubiquitous nature of the connection, which ranges from mitochondrial aspects in the prenatal and environmental risk factors/exposures [2,3,8] to biomarkers/metabolites [5,8,12,13,14,15,16], enzymology [12], and the positive response to therapy, at least in part targeting the mitochondria [8,11,12,15,16,18,19].

Many papers also relate to another potential etiology of ASD, particularly the immune system, in which multiple interactive domains are highlighted, including innate immunity, immune deficiency, autoimmunity, inflammation, and mast cell activation [7,8,10,11,15,16]. However, similarly to neurons, leukocytes are highly dependent on energy metabolism, and the connections between energy and immunity are many and important. In one example, folate transport to brain is affected predominately by autoimmunity and mitochondrial function [16].

Is ASD itself a result of abnormal mitochondria, with effects on neurons and/or leukocytes—essentially a “Bad Trio” of mitochondrial dysfunction, oxidative stress and redox regulation, and immune system dysfunction [3]? Is there a room for a primary role in pathogenesis for other pathways, including synapses [6,7,8], methylation [8,18], autonomic nervous function [9,20], ion channels [6], hypermobility [9], and microbiome [14], at least in some cases? Or, perhaps, do these concepts work within the mitochondrial hypothesis? For example, synapses have high-energy requirements and have high densities of mitochondria. Folate and cobalamin, important cofactors in methylation, are also important cofactors in energy metabolism. Similar to all neurons, autonomic neurons have high energy requirements. Leaking ion channels require ATP-driven pumps to reestablish homeostasis placing additional pressure on energy metabolism. Joint mobility is dependent on muscle tone, which is yet another tissue with high-energy requirements. Additionally, the microbiome produces metabolites that cross over into the body and likely interact with mitochondrial metabolism of the host, especially if the blood–brain barrier is compromised. In any event, each of these concepts, from synapses to microbiome, are important to include in further research and to consider in a personalized medicine approach for the diagnosis and treatment in the individual ASD patient.

If mitochondria are the key to ASD, why is it that the vast majority of ASD patients that have undergone whole genome sequencing (WGS) have yet to have identifiable pathogenic variants in genes involved in mitochondrial function? Certainly, people with mitochondrial disease (a primary genetic mutation affecting energy metabolism) often have ASD, and many people with ASD do have mitochondrial disease, but this is a minority per our present understanding. Perhaps the answer is in the combined effects of multiple genetic variants, each one benign in of itself and oftentimes common [7]. Perhaps the presence of primary mutations in many different pathways among people with ASD, in hundreds of different genes suggests that abnormal mitochondria are rarely the cause of ASD, but rather an important downstream mechanism in the common pathophysiology of ASD. Thus, mitochondrial dysfunction may be acquired in most people with ASD [3]. Perhaps the answer is a little of both as well as other mechanisms not considered herein or even not yet proposed. Further research is needed to examine these possibilities.

Effective treatment and management for individuals with ASD is only starting to be uncovered, but we believe the articles within this volume provide insight and a starting point for the evolution of rational and optimal treatments for individuals with ASD.

## Data Availability

Not applicable.

## References

[1] Bokadia H., Rai R., Torres E.B. (2020). Digitized ADOS: Social Interactions beyond the Limits of the Naked Eye. J. Pers. Med..

[2] Grivas G., Frye R., Hahn J. (2021). Pregnant Mothers’ Medical Claims and Associated Risk of Their Children being Diagnosed with Autism Spectrum Disorder. J. Pers. Med..

[3] Frye R., Cakir J., Rose S., Palmer R., Austin C., Curtin P., Arora M. (2021). Mitochondria May Mediate Prenatal Environmental Influences in Autism Spectrum Disorder. J. Pers. Med..

[4] Ferreira M., Loyacono N. (2021). Rationale of an Advanced Integrative Approach Applied to Autism Spectrum Disorder: Review, Discussion and Proposal. J. Pers. Med..

[5] Mesleh A.G., Abdulla S.A., El-Agnaf O. (2021). Paving the Way toward Personalized Medicine: Current Advances and Challenges in Multi-OMICS Approach in Autism Spectrum Disorder for Biomarkers Discovery and Patient Stratification. J. Pers. Med..

[6] Calderoni S., Ricca I., Balboni G., Cagiano R., Cassandrini D., Doccini S., Cosenza A., Tolomeo D., Tancredi R., Santorelli F.M. (2020). Evaluation of Chromosome Microarray Analysis in a Large Cohort of Females with Autism Spectrum Disorders: A Single Center Italian Study. J. Pers. Med..

[7] Vanzo R.J., Prasad A., Staunch L., Hensel C.H., Serrano M.A., Wassman E.R., Kaplun A., Grandin T., Boles R.G. (2021). The Temple Grandin Genome: Comprehensive Analysis in a Scientist with High-Functioning Autism. J. Pers. Med..

[8] Way H., Williams G., Hausman-Cohen S., Reeder J. (2021). Genomics as a Clinical Decision Support Tool: Successful Proof of Concept for Improved ASD Outcomes. J. Pers. Med..

[9] Casanova E., Baeza-Velasco C., Buchanan C., Casanova M. (2020). The Relationship between Autism and Ehlers-Danlos Syndromes/Hypermobility Spectrum Disorders. J. Pers. Med..

[10] Rossignol D., Frye R. (2021). A Systematic Review and Meta-Analysis of Immunoglobulin G Abnormalities and the Therapeutic Use of Intravenous Immunoglobulins (IVIG) in Autism Spectrum Disorder. J. Pers. Med..

[11] Theoharides T.C. (2021). Ways to Address Perinatal Mast Cell Activation and Focal Brain Inflammation, including Response to SARS-CoV-2, in Autism Spectrum Disorder. J. Pers. Med..

[12] Barone R., Bastin J., Djouadi F., Singh I., Karim M., Ammanamanchi A., McCarty P., Delhey L., Shannon R., Casabona A. (2021). Mitochondrial Fatty Acid β-Oxidation and Resveratrol Effect in Fibroblasts from Patients with Autism Spectrum Disorder. J. Pers. Med..

[13] McCarty P.J., Pines A.R., Sussman B.L., Wyckoff S.N., Jensen A., Bunch R., Boerwinkle V.L., Frye R.E. (2021). Resting State Functional Magnetic Resonance Imaging Elucidates Neurotransmitter Deficiency in Autism Spectrum Disorder. J. Pers. Med..

[14] Qureshi F., Adams J., Hanagan K., Kang D.-W., Krajmalnik-Brown R., Hahn J. (2020). Multivariate Analysis of Fecal Metabolites from Children with Autism Spectrum Disorder and Gastrointestinal Symptoms before and after Microbiota Transfer Therapy. J. Pers. Med..

[15] Bobrowski-Khoury N., Ramaekers V., Sequeira J., Quadros E. (2021). Folate Receptor Alpha Autoantibodies in Autism Spectrum Disorders: Diagnosis, Treatment and Prevention. J. Pers. Med..

[16] Rossignol D.A., Frye R.E. (2021). Cerebral Folate Deficiency, Folate Receptor Alpha Autoantibodies and Leucovorin (Folinic Acid) Treatment in Autism Spectrum Disorders: A Systematic Review and Meta-Analysis. J. Pers. Med..

[17] Pines A.R., Sussman B., Wyckoff S.N., McCarty P.J., Bunch R., Frye R.E., Boerwinkle V.L. (2021). Locked-in Intact Functional Networks in Children with Autism Spectrum Disorder: A Case-Control Study. J. Pers. Med..

[18] Rossignol D.A., Frye R.E. (2021). The Effectiveness of Cobalamin (B12) Treatment for Autism Spectrum Disorder: A Systematic Review and Meta-Analysis. J. Pers. Med..

[19] Adams J.B., Bhargava A., Coleman D.M., Frye R.E., Rossignol D.A. (2021). Ratings of the Effectiveness of Nutraceuticals for Autism Spectrum Disorders: Results of a National Survey. J. Pers. Med..

[20] Foldes S.T., Jensen A.R., Jacobson A., Vassall S., Foldes E., Guthery A., Brown D., Levine T., Tyler W.J., Frye R.E. (2021). Transdermal Electrical Neuromodulation for Anxiety and Sleep Problems in High-Functioning Autism Spectrum Disorder: Feasibility and Preliminary Findings. J. Pers. Med..

[21] Musetti A., Manari T., Dioni B., Raffin C., Bravo G., Mariani R., Esposito G., Dimitriou D., Plazzi G., Franceschini C. (2021). Parental Quality of Life and Involvement in Intervention for Children or Adolescents with Autism Spectrum Disorders: A Systematic Review. J. Pers. Med..

